# Nicotinamide Mononucleotide Administration Prevents Doxorubicin-Induced Cardiotoxicity and Loss in Physical Activity in Mice

**DOI:** 10.3390/cells12010108

**Published:** 2022-12-27

**Authors:** Marielle Margier, Chisaka Kuehnemann, Nicolas Hulo, Jazmin Morales, Prasanna Vadhana Ashok Kumaar, Cecile Cros, Helene Cannelle, Julie Charmetant, Eric Verdin, Matthias Canault, Alessia Grozio

**Affiliations:** 1 LGD SARL, 13880 Velaux, France; 2 Buck Institute for Research on Aging, Novato, CA 94945, USA; 3 Nuvamid SA, 1260 Nyon, Switzerland; 4 Department of Medicine, University of California at San Francisco, San Francisco, CA 94143, USA

**Keywords:** doxorubicin, nicotinamide mononucleotide, NAD+, cardiotoxicity, physical activity impairment, oxidative stress, p53-mediated pathways, inflammation, mitochondrial functions

## Abstract

Doxorubicin (Doxo) is a widely used antineoplastic drug with limited clinical application due to its deleterious dose-related side effects. We investigated whether nicotinamide mononucleotide (NMN) could protect against Doxo-induced cardiotoxicity and physical dysfunction in vivo. To assess the short- and long-term toxicity, two Doxo regimens were tested, acute and chronic. In the acute study, C57BL6/J (B6) mice were injected intraperitoneally (i.p.) once with Doxo (20 mg/kg) and NMN (180 mg/kg/day, i.p.) was administered daily for five days before and after the Doxo injection. In the chronic study, B6 mice received a cumulative dose of 20 mg/kg Doxo administered in fractionated doses for five days. NMN (500 mg/kg/day) was supplied in the mice’s drinking water beginning five days before the first injection of Doxo and continuing for 60 days after. We found that NMN significantly increased tissue levels of NAD+ and its metabolites and improved survival and bodyweight loss in both experimental models. In addition, NMN protected against Doxo-induced cardiotoxicity and loss of physical function in acute and chronic studies, respectively. In the heart, NMN prevented Doxo-induced transcriptomic changes related to mitochondrial function, apoptosis, oxidative stress, inflammation and p53, and promyelocytic leukemia nuclear body pathways. Overall, our results suggest that NMN could prevent Doxo-induced toxicity in heart and skeletal muscle.

## 1. Introduction

According to the American Cancer Society (ACS) there are more than 18 million cancer survivors existing in the United States as of 2022 and this number is projected to increase to 22.2 million by 2030 [1]. Studies among long-term survivors of childhood cancer suggest that most of the anti-cancer treatments such as chemotherapeutic drugs cause a range of toxic side effects, which can occur even long after treatment, that resemble pathologies associated with aging, including cardiomyopathies, secondary tumors, fatigue and physical and cognitive impairments [2,3,4,5,6].

Doxorubicin (Doxo), an anthracycline antibiotic, is the first-line chemotherapeutic drug used for various hematological malignancies and solid tumors. However, the use of Doxo is limited by its dose-dependent cardiac and muscular toxic effects that lead to cardiac dysfunctions [7], skeletal muscle weakness [8] and severe fatigue [9]. Mechanistically, oxidative stress has been ascribed as one of major contributors to Doxo-induced cellular toxicity which causes mitochondrial dysfunction and DNA damage, ultimately leading to cellular apoptosis/necrosis [10,11,12]. Moreover, it has been shown that Doxo induces cellular senescence with a senescence-associated secretory phenotype (SASP) in no tumor-bearing mice [13].

To date, effective strategies to prevent or treat chemotherapy-induced cardiomyopathy, fatigue and skeletal muscle weakness are missing.

Since many anti-cancer therapies appear to promote a premature aging-like state, interventions that target fundamental aging pathways may ameliorate the late complications in cancer survivors.

Nicotinamide adenine dinucleotide (NAD+) is a pivotal redox cofactor for metabolism and ATP production and a key substrate for NAD+-consuming enzymes, including sirtuins (SIRTs), poly-ADP-ribose polymerases (PARPs), CD38/157 ectoenzymes that are involved in healthspan and longevity regulation [14,15]. Mounting evidence links a compromised NAD+ status to the hallmarks of aging and to the exacerbation of age-associated diseases such as metabolic syndrome, diabetes, neurodegeneration and chronic inflammation [15,16].

In mammals, NAD+ biosynthesis mostly relies on the salvage pathway in which nicotinamide mononucleotide (NMN) is a fundamental intermediate. NMN is synthesized from nicotinamide, a form of vitamin B3 and 5′-phosphoribosyl-1-pyrophosphate (PRPP), by the rate-limiting enzyme, nicotinamide phosphoribosyl transferase (NAMPT). NMN is subsequently converted into NAD+ by NMN adenylyl transferases (NMNATs). Moreover, NAD+ can be also generated through the de novo pathway from tryptophan in specific tissue such as the liver [17].

Aside for the beneficial effects demonstrated in age-associated diseases [18], boosting NAD+ levels by the systemic administration of NMN has shown to protect aged kidney from both ischemia–reperfusion and cisplatin-induced acute kidney injuries [19], cochlear cells from cisplatin-induced ototoxicity [20], aged heart from myocardial ischemia/reperfusion injury [21,22] and to prevent cisplatin-induced cognitive impairments [23] in rodents. Moreover, NMN in combination with troxerutin has been shown to protect cardiac tissue against Doxo treatment by enhancing mitochondrial function [24]. Taken together, these observations suggest that the augmentation of NAD+ levels might prevent the short-term and long-term toxic side effects of chemotherapeutic treatment. Therefore, this study was designed to investigate whether NMN administration protects against Doxo-induced cardiotoxicity and loss of physical activity in vivo and to determine the underlying molecular mechanisms by performing an extensive transcriptomic analysis in tissue.

## 2. Materials and Methods

### 2.1. Animal Experiments

Two different Doxo regimes, acute and chronic, have been implemented to assess the cardiotoxicity and skeletal muscles functionality.

Animal experiments were approved by the Animal Care and Ethic Committee of the CREFRE (Centre régional d’exploration fonctionnelle et de ressources expérimentales)/Inserm US 006 and the Institutional Animal Care and Use Committee (IACUC) of the Buck Institute (Novato, CA, USA). For the acute toxicity study, male C57BL6/J (B6) mice were purchased from Janvier SAS (St Berthevien, France) at 9 weeks of age and acclimated for at least five days before any drug administration. Standard chow diet (SAFE^®^ 150, Augy, France; 18% protein rodent diet) and tap water were provided ad libitum. Animals were housed (4–6 mice/cage) on a 12 h light-dark cycle at 22 ± 2 °C and 55 ± 10% relative humidity. For the chronic toxicity study, male B6 mice were purchased from Jackson Laboratories (Bar Harbor, ME) at 12 weeks of age (RRID: IMSR_JAX:000664) and acclimated for at least two weeks before any treatment. Standard chow diet (Teklad global, 18% protein rodent diet, 2918) was provided ad libitum and mice were housed (4 mice/cage) on a 12 h light-dark cycle at 22 ± 2 °C and 55 ± 10% relative humidity.

### 2.2. Doxorubicin and NMN Administration

For the acute toxicity study, Doxo was administered by a single intraperitoneal (i.p.) injection at 20 mg/kg body weight (BW) (Abcam, Cambridge, UK, Product# ab120629) [25]. Briefly, Doxo was prepared at 2 mg/mL in saline (NaCl 0.9%) (Fisher Scientific, Pittsburgh, PA, USA, Product# 10356340) and the volume of administration was 10 mL/kg. Control mice received the same saline volume by i.p. injections. Treatment with NMN (Seneque^®^, St. Priest, France) was administered by i.p. injection at 180 mg/kg/day (mouse body weight) and was initiated 5 days before Doxo injection and for an additional 5 days. The day of Doxo administration, NMN treatment was injected 30 min before Doxo. The NMN powder was dissolved in saline (vehicle) and extemporaneously prepared. Doxo-treated mice were injected either with vehicle (Doxo) or NMN (Doxo-NMN) whereas control mice (Ctrl) received vehicle only for the entire duration of the study. Thirty-nine mice for Ctrl, 71 mice for Doxo and 46 mice for Doxo-NMN were enrolled in the acute toxicity study. Three cohorts were generated as follows: Cohort-1: Ctrl mice (n = 17), Doxo mice (n = 23) and Doxo-NMN mice (n = 16); Cohort-2: Ctrl mice (n = 12), Doxo mice (n = 24) and Doxo-NMN mice (n = 16); Cohort-3: Ctrl mice (n = 10), Doxo mice (n = 24) and Doxo-NMN mice (n = 14).

Survival rate was assessed throughout the experimental phase and the body weights were recorded on the day of Doxo administration (D0) and 5 days later (D5) on all three cohorts. Blood was collected in EDTA containing tubes through the retro-orbital sinus 5 days after Doxo administration (D5) (2 h after the last NMN injection). LC-MS analysis of NMN, NAD+ was performed on blood from cohort-2 and related metabolites on blood from cohort-1. Dosage of lactate dehydrogenase (LDH) was performed on plasma from all the three cohorts and troponin-I on plasma from cohort-1 and 2. Cardiac function was assessed by echocardiography at D5 on all the three cohorts. Finally, all cohorts were euthanized at D5 and tibia were collected and measured and hearts were collected 2 h after the last injection of NMN. NMN, NAD+ and related metabolites dosages were performed on hearts of cohort-2. RNA-seq analysis was performed on 13–14 hearts for each group of cohort-1.

For the chronic toxicity study, Doxo was administered as previously described [26]. Briefly, male B6 mice at 14–16 weeks of age were injected i.p. with 4 mg/kg BW/day of Doxo (Tocris Bioscience, Product# 2252) five times for a cumulative dose of 20 mg/kg within two weeks (three times, every other day in the first week and two times, every other day in the second week). Endotoxin-free PBS (EMD Millipore, Product# TMS-012-A) was used as the diluent. Control mice received the same volume of endotoxin-free PBS by i.p. injections. Water consumption was measured for one week prior to the beginning of NMN administration. NMN (Seneque^®^, St. Priest, France) was then administered in drinking water ad libitum at 500 mg/kg BW/day, based on the previously measured water consumption and BW. The administration started five days prior to the first Doxo injection and continued for an additional 60 days. BW and water intake were monitored twice per week, at the same time each week and NMN concentration in drinking water was adjusted to account for water intake and BW change. The NMN solution was prepared weekly in small batches by dissolving NMN into autoclaved drinking water and filtered sterile. Water bottles containing NMN were changed twice weekly for the entire study. Doxo-treated mice either received regular drinking water (Doxo) or 500 mg/kg/day NMN in drinking water (Doxo-NMN), whereas control mice (Ctrl) received regular drinking water for the entire duration of the study. Twenty-four mice for Ctrl, 28 mice for Doxo and 28 mice for Doxo-NMN were used in this study. Three cohorts were generated as follows: Cohort-1: Ctrl mice (n = 8), Doxo mice (n = 8) and Doxo-NMN (n = 8); Cohort-2: Ctrl mice (n = 8), Doxo mice (n = 8) and Doxo-NMN (n = 8); Cohort-3: Ctrl mice (n = 8), Doxo mice (n = 12) and Doxo-NMN (n = 12). All cohorts were euthanized after 60 days from the first Doxo injection and tissue were collected.

BW measurement, survival rate and the measurement of cytokines and chemokines in plasma were assessed for all three cohorts. Echocardiographic parameters were measured on cohort-1 and cohort-2, body composition was assessed on cohort-2 and cohort-3. Exercise capacity by treadmill was performed on cohort-2 and rotarod analysis on cohort-3. LC-MS analysis of NAD+ and NAD-related metabolites was performed on blood and hearts of cohort-1 and 2 and gastrocnemius muscles of cohort-3. RNA-seq analysis was performed on 5 or 6 hearts for each group of cohort-3. The determination of 4-HNE protein adducts and the expression of senescence markers was performed on gastrocnemius muscle of cohort-1 and -2 and of cohort-1, -2 and -3, respectively.

### 2.3. Body Composition

Measurements of fat and lean mass were acquired in live mice using the InAlyzer Dual Energy X-ray Absorption (DEXA) body composition analyzer (Medicors) at 50 days after the first Doxo injection.

### 2.4. Transthoracic Echocardiography

Echocardiographic analysis of mice in the acute toxicity study was performed at Cardiomedex laboratory (Escalquens, France). Animals were placed on a heating pad (37 °C) and were anesthetized under isoflurane (1.5–2%). Echocardiography was performed using a high-frequency 16-MHz linear transducer (VF16-5 probe, Siemens Healthineers) connected to an ultrasound system (Acuson NX3 Elite, Siemens Healthineers). For the chronic toxicity study, two-dimensional (2D) transthoracic echocardiographic analysis was performed at 53 days after the first Doxo injection by the mouse phenotyping core at Buck institute using a high frequency (20–46 MHz) Visualsonics Vevo 3100 micro-ultrasound system with the echocardiography MX-400 probe, (Visualsonics). Individual mice were placed on a heating pad (37 °C) and minor sedation (1.5–2% isoflurane oxygen) was used to anesthetize the animal during the procedure.

Data acquisition was performed 2D-, M- and Doppler modes, from parasternal long- and short axis, as well as apical 4 chamber views. Left ventricle volumes, diameters and wall thicknesses were measured using Compacs software (Siemens) or VevoLab software (Visualsonics). The LV ejection fraction (EF) and fractional shortening (FS) were calculated with the following formulas:

EF% = [end-diastolic volume (EDV) − end-systolic volume]/EDV × 100;

FS% = [left ventricle internal diameter in diastole (LVIDd) − left ventricle internal diameter in systole]/LVIDd × 100.

### 2.5. Treadmill Exhaustion Test

For the chronic toxicity study, the exercise capacity was assessed by using a motorized treadmill (PanLab, Harvard Lab Apparatus) at 45 days after the first Doxo injection. The mice received training twice in the week leading up to the test. On the test day, mice ran on the treadmill at an initial speed of 16.67 m/min at a fixed slope of 10% for 5 min, after which the speed was increased by 3.33 m/min every subsequent 2 min until the mice were exhausted. Mice were considered to be exhausted if they remained in the fatigue zone for 5 continuous seconds despite mechanical prodding, at which point, they were promptly removed from the treadmill and running time and distance recorded. Work and power were calculated as previously described [27]. Work (J) = body mass (kg) × gravity (9.81 m/s^2^) × vertical speed (m/s × angle) × time (s). Power (W) = work (J)/time (s).

### 2.6. Rotarod Test

For the chronic toxicity study, motor coordination and endurance were assessed by measuring latency to fall off from an accelerating rotarod device (Ugo Basile, model 47600) at 45 days after the first Doxo injection. Mice were trained twice in the week leading up to the test. On the first day of training, the mice were placed on the rotarod for 5 min at a constant speed of 5 RPM, repeating for three trials. For the second training and test day, speed was gradually ramped up from 5 RPM to 30 RPM over the course of 240 s and the maximal latency set at 360 s per trial. Mice that were still able to maintain their balance after 360 s were removed from the rotarod and returned to their cages. The time to latency averaged across three trials performed consecutively.

### 2.7. Liquid Chromatography Mass Spectrometry (LC-MS) Analysis (NAD+ Metabolome)

For the acute toxicity study, NAD+ and related metabolites were extracted from blood and pre-weighed hearts by different methods. To measure the levels of NAD+ and NMN, the sample preparation procedure was performed as previously described [28]. To measure the levels of nicotinic acid adenine dinucleotide (NAAD), nicotinamide (NAM), 1-methylnicotinamide (n-MetNAM) and methyl-2-pyridone-5-carboxamide (Me2Py), blood sample preparation procedure was performed as previously described [29], and heart sample preparation was performed as the chronic toxicity study (see the next paragraph). All metabolomic data was accomplished using a XEVO TQD mass spectrometer coupled to an Acquity UPLC H-Class plus system (Waters). NAD+ and NMN were separated using a Acquity HSS T3 C18 column (2.2 × 150 mm × 1.8 μm) kept at a constant temperature (30 °C). Mobile phase A consisted of 0.01% Difluoroacetic Acid (DFA) and mobile phase B was 100% acetonitrile. The following linear gradient was run for 11 min at a flow rate of 0.45 mL/min: 0–1 min 100% A, 2.5 min 97.5% A, 7 min 5% A, 8.1 min 100% B. Standards were prepared in perchloric acid and samples were kept at 5 °C in the autosampler tray prior to injection. Source parameters were set as follows: capillary 2000 V, source temperature 500 °C, cone source voltage 10 V, cone source gas flow 50 L/h, desolvation gas flow 1000 L/h. Data acquisition was optimized in positive ion mode using multiple reaction monitoring (MRM). Transitions monitored were as follows: NAD+ 332.76–136.01 (cone voltage = 20 V, collision energy = 58 V) and NMN 335.03–96.96 (cone voltage = 24 V, collision energy = 26 V). NAAD, NAM, n-MetNAM and Me2Py were separated using an Atlantis Hilic Silica column (4.6 × 100 mm × 3 µm), kept at a constant temperature (25 °C). Mobile phase A consisted of 5 mM ammonium formate, 0.1% formic acid in water/acetonitrile (20/80) and mobile phase B was 5 mM ammonium formate, 0.1% formic acid in water/acetonitrile (50/50). The following isocratic gradient was run for 32 min at a flow rate of 1 mL/min: 0–1 min 100% A, 1.01–2.7 min 100% B and 2.71–32 min 100% A. Standards were prepared in the mobile phase A and samples were kept at 5 °C in the autosampler tray prior to injection. The source parameters were set as follows: capillary 3000 V, source temperature 250 °C, cone source voltage 20 V, cone source gas flow 32 L/h, desolvation gas flow 1000 L/h and. Data acquisition was optimized in positive mode using MRM. Transitions monitored were as follows: NAAD 665.07–136.06 (cone voltage = 32 V, collision energy = 58 V), NAM 122.96–53 (cone voltage = 40 V, collision energy = 58 V), n-MetNAM 136.98–78.05 (cone voltage = 42 V, collision energy = 24 V) and Me2Py 153.0–77.99 (cone voltage = 50 V, collision energy = 28 V). MassLynx software was used for mass spectrometry data acquisition and TargetLynx software was used for analysis (Waters). Relative levels for the metabolites were determined using area response of the targeted metabolites. Quantification was done based on a calibration curve with commercial metabolite standards.

For the chronic toxicity study, NAD+ and related metabolites were extracted from frozen pre-weighed tissues collected at day 60 after the first Doxo injection with 80% methanol containing internal standard mix (13C NAD+ and 13C NAM, Cambridge Isotope Laboratories). Briefly, frozen tissue was homogenized in a bullet blender (Next Advance bead homogenizer) in 80% methanol for 2 cycles of 2 min. Following complete homogenization, samples were vortexed for 10 sec and incubated for 30 min on ice. Upon centrifugation at 18,000 rcf for 10 min, the supernatant was dried in a speed vacuum. The final dried pellet was resuspended in starting buffer conditions for subsequent LC-MS analysis. For analysis of metabolites from whole blood, 0.5 mL methanol was added to 100 µL frozen whole blood collected by cardiac puncture at day 60 after the first Doxo injection and intermittently vortexed to allow simultaneous thawing and extraction. After 15 min, the samples were centrifuged and processed in the same way as the tissues. Targeted, LC-MS-based metabolomics for the measurement of NAD+ metabolites was performed as previously described [30]. Metabolomics data acquisition was accomplished using a Q Exactive Mass Spectrometer coupled to a Vanquish LC system (Thermo Scientific). Separation of NAD+ and related metabolites was achieved using a Hypercarb column (5 mm, 100 × 2.1 mm column, Thermo Scientific) operating at 60 °C. Mobile phase A consisted of 7.5 mM ammonium acetate with 0.05% (*v*/*v*) ammonium hydroxide and solvent B was 0.05% (*v*/*v*) ammonium hydroxide in acetonitrile. The following linear gradient was run for 12 min at a flow rate of 0.250 mL/min: 0–1 min 5% B, 6 min 60% B, 6.1–7.5 min 90% B, 7.6–12 min 5% B. The source parameters in Q Exactive MS optimized by direct infusion of NAD+ metabolites were as follows: sheath gas flow rate 45, aux gas flow rate 2, spray voltage 3.75 kV, capillary temperature 320 °C, S-lens RF level 55 and probe temperature 120 °C. Data acquisition was optimized in positive mode FullMS1-ddMS2 (Top4) and coupled targeted single and parallel ion monitoring (tSIM-PRM) within a scan range of 50–750 *m*/*z*. Metabolite identification was confirmed with MS1/MS2 spectra match and retention time match with standards run in parallel. Characteristic fragments of NAD+ and related metabolites were confirmed with online metabolome databases such as Metlin and Massbank. Normalized area under the curve (AUC) for each metabolite was derived from MS1 extracted ion chromatograms. Data analysis was performed using Freestyle and Excalibur Quan Browser. Relative levels for the metabolites were determined using area response ratios of the targeted metabolites and the corresponding heavy labeled internal standard. Absolute quantification was done based on a 10-point calibration curve with commercial metabolite standards.

### 2.8. Troponin-1, Lactate Dehydrogenase, 4-Hydroxynonenal and Cytokines and Chemokines Analysis

The levels of troponin-I and lactate dehydrogenase (LDH) were measured in plasma using a troponin-I ELISA kit (Mybiosource, Product# MBS280167) and a LDH colorimetric kit (Horiba Medical, Product# A11A01824), respectively, according to the manufacturer’s protocol.

The levels of 4-HNE (4-Hydroxynonenal) protein adducts in gastrocnemius muscle lysates were measured using a 4-HNE ELISA kit (Abcam, Product# ab238538) according to the manufacturer’s protocols. 4-HNE quantification was normalized to proteins content in homogenates using the Pierce BCA protein assay kit (Thermo Fisher Scientific, Product# 23227).

Plasma was collected by tail bleeding in EDTA-treated tubes at 59 days after the first Doxo injection from each group in the chronic study and 18 different cytokines and chemokines were quantified simultaneously using the Mouse High Sensitivity T Cell Discovery Array 18-plex (MDHSTC18) from Eve Technologies Corp (Calgary, Canada).

### 2.9. RNA-seq Analysis in Heart Tissue

The frozen heart samples from the acute and chronic toxicity studies were submitted to Genewiz (Leipzig, Germany) or Azenta (former Genewiz, South Plainfield, NJ, USA), respectively. Sequencing was performed on an Illumina NovaSeq™ 6000 platform after total RNA extraction, poly(A) selection and library generation. Approximately 30 million single-end reads of 150 bp per sample were obtained and the quality of reads was checked with the fastqc tool. Sequences were mapped against the Mouse Reference Genome (assembly Mm10, UCSC, December 2011) with the STAR program (version 2.7.0) [31] and count tables containing the number of mapped reads per gene were produced with featureCounts (version 2.0.0). Count tables were then imported into R to do the differential expression analysis with edgeR (version 3.32.1) [31]. Library sizes were adjusted with a scaling factor calculated using a trimmed mean of M-values (TMM) between each pair of samples. The common dispersion and tagwise dispersions were estimated with the estimateDisp function. After negative binomial glm fitting the quasi-likelihood (QL) F-test was applied for the testing procedure. The order differentially expressed gene lists obtained with this procedure were then used to do gene set enrichment analysis (GSEA) on various pathway databases with clusterProfiler functions (version 3.18.1) [32]. Genes of interest were visualized with heatmap either generated with ggplot2 (version 3.3.5) or with ComplexHeatmap (version 2.6.2) when genes and samples were clustered. Pathways significantly enriched (q value < 0.02) were visualized with ggplot2 scripts. Redundant pathways and pathways irrelevant for a given tissue were removed.

### 2.10. Quantitative RT-PCR (RT-qPCR)

Total RNA was extracted using PureLink™ RNA Mini Kit (Thermo Fisher scientific, Product# 12183025), then reverse transcribed using High-Capacity cDNA Reverse Transcription Kit (Thermo Fisher scientific, Product# 4368814). RT-qPCR reactions were performed using SensiFAST™ Probe No-ROX Kit (Thomas Scientific, Product# C755H92). The amounts of mRNA were normalized relative to Beta -actin mRNA (Thermo Fisher scientific, Product# 4352341E). Specific primer sequences used for RT-qPCR are p16^INK4a^ (p16) (Forward 5′-AACTCTTTCGGTCGTACCCC-3; Reverse 5′-TCCTCGCAGTTCGAATCTG-3′) and for p21^CIP1^(p21) (Forward 5′-TTGCCAGCAGAATAAAAGGTG-3′; Reverse 5′-TTTGCTCCTGTGCGGAAC-3′).

### 2.11. Statistical Analysis

All values are presented as mean values ± standard error of the mean. Outlier identification and statistical analyses were performed using GraphPad Prism software, version 9.3.0 (GraphPad Software Inc., San Diego, CA, USA). Differences between more than two groups of unpaired data were tested by parametric one-way ANOVA followed by Tukey’s post hoc test or nonparametric Kruskal–Wallis test followed by Dunnett’s post hoc test. Differences between two groups were assessed using parametric unpaired, two-tailed t test. The Mantel–Cox log rank test was performed to compare the survival of the different groups. Values of *p* < 0.05 were considered significant. * *p* < 0.05, ** *p* < 0.01, *** *p* < 0.001, **** *p* < 0.0001.

## 3. Results

### 3.1. NMN Improves Survival, Body Weight Loss and Cardiac Function Decline Induced by the Acute Doxo Treatment in Mice

**Figure 1:**
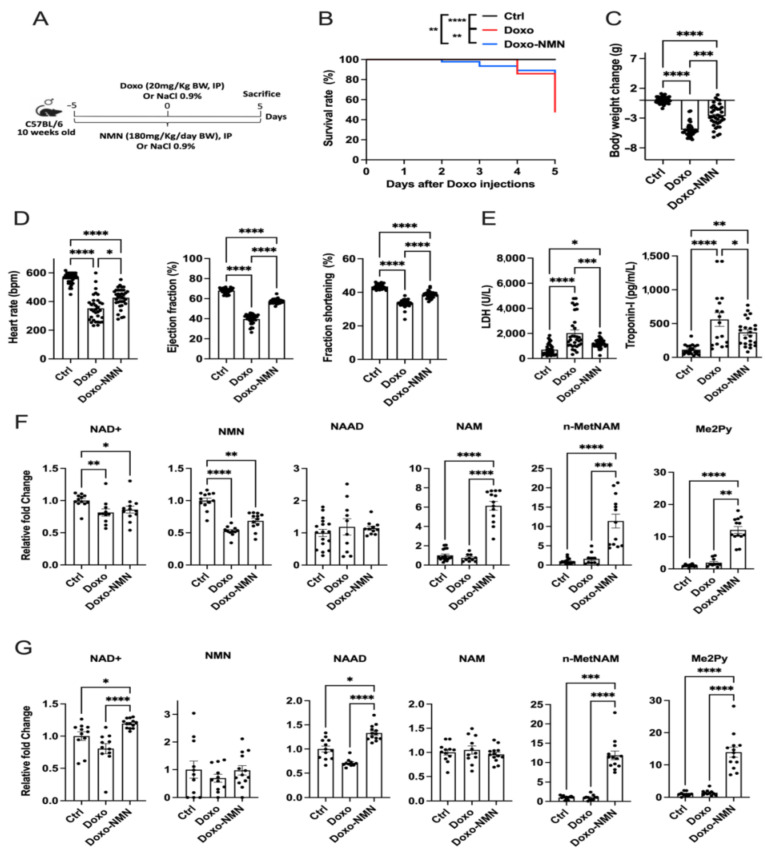
Treatment of NMN improves survival, body weight loss and cardiac function impaired by acute Doxo toxicity and affects the NAD+ metabolome in their bloodstream and heart. (**A**) Study design for the acute regime in Doxo-induced cardiotoxicity mouse model. Treatment with NMN (180 mg/kg/day, i.p.) was initiated 5 days before the Doxo injection (20 mg/kg, i.p.) and continued over 5 days. Survival rate, body weight variation and cardiac function were assessed as described in methods. (**B**) Survival curve (percentage, %) of Ctrl, Doxo and Doxo-NMN mice. Log-rank (Mantel–Cox) test. ** *p* < 0.01, **** *p* < 0.0001. (**C**) Body weight variation at the end of the treatment period. Body weight variation has been calculated as follows: Body weight at the day of sacrifice (day 5)—body weight at the day of Doxo-injection (day 0). Nonparametric Kruskal–Wallis test followed by Dunn’s post-test. *** *p* < 0.001, **** *p* < 0.0001. (**D**) Cardiac function at the end of the treatment period. Heart rate, ejection fraction and fraction shortening were assessed by echocardiography on day 5. Nonparametric Kruskal–Wallis test followed by Dunn’s post-test. * *p* < 0.05, **** *p* < 0.0001. (**E**) LDH and troponin–-I levels at the end of the treatment period. One-way Anova followed by Tukey’s post-test. * *p* < 0.05, ** *p* < 0.01 *** *p* < 0.001, **** *p* < 0.0001. (**F**) Levels of NAD+, NMN, NAAD, NAM, n-MetNAM and Me2Py (relative to Ctrl group) in blood on day 5. Nonparametric Kruskal–Wallis test followed by Dunn’s post-test were used for NAD+, NMN, NAM, n-MetNAM and Me2Py and One-way Anova followed by Tukey’s post-test were used for NAAD. * *p* < 0.05, ** *p* < 0.01, *** *p* < 0.001, **** *p* < 0.0001. (**G**) Levels of NAD+, NMN, NAAD, NAM, n-MetNAM and Me2Py (relative to Ctrl group) in heart on day 5. Nonparametric Kruskal–Wallis test followed by Dunn’s post-test were used for NAD+, NAAD and n-MetNAM and One-way Anova followed by Tukey’s post-test were used for NMN, NAM and Me2Py. * *p* < 0.05, *** *p* < 0.001, **** *p* < 0.0001. Data are shown as means ± the standard error.

To investigate whether NMN treatment could reduce the Doxo-induced cardiotoxicity, male C57BL/6 mice were administered NMN (180 mg/kg/day, i.p.) or saline once daily, 5 days prior Doxo injection (20 mg/kg, i.p.) and maintained for an additional 5 days (Figure 1A). Survival rate was assessed throughout the experimental phase. At D5, all the mice in the Ctrl group survived. Doxo-treatment resulted in a significant decrease in mouse survival (48%). Administration of NMN (Doxo-NMN group) prevented Doxo-induced death and significantly improved survival at D5 post-Doxo injection up to 80% (Figure 1B). Accordingly, a significant reduction in body weight was noticed at D5 in Doxo mice compared to the Ctrl group (−0.0 ± 0.1 g (Ctrl) vs. −5.0 ± 0.2 g (Doxo) *p* < 0.0001). NMN treatment significantly minimized the Doxo-induced body weight loss but did not fully prevent it (−2.7 ± 0.3 g (Doxo-NMN), *p* < 0.001 Doxo vs. Doxo-NMN and *p* < 0.0001 Ctrl vs. Doxo-NMN) (Figure 1C). Cardiac function was then evaluated at D5 through echocardiography and plasma LDH and troponin-I level assessments. As compared to the Ctrl mice, Doxo injection resulted in a major drop in heart rate (HR), ejection fraction (EF) and fractional shortening (FS) (HR: 558.0 ± 7.0 bpm (Ctrl) vs. 352.8 ± 15.7 bpm (Doxo), *p* < 0.0001; EF: 68.0 ± 0.4% (Ctrl) vs. 39.6 ± 0.8% (Doxo), *p* < 0.0001; FS: 43.5 ± 0.2% (Ctrl) vs. 33.2 ± 0.4% (Doxo), *p* < 0.0001) (Figure 1D), indicating an important alteration of the cardiac function. Treatment with NMN has significantly attenuated these deleterious effects although did not completely prevent them (HR: 426.6 ± 11.2 bpm (Doxo-NMN), *p* < 0.05 Doxo vs. Doxo-NMN and *p* < 0.0001 Ctrl vs. Doxo-NMN; EF: 57.5 ± 0.5% (Doxo-NMN), *p* < 0.0001 Doxo vs. Doxo-NMN and *p* < 0.0001 Ctrl vs. Doxo-NMN; FS: 38.6 ± 0.3% (Doxo-NMN), *p* < 0.0001 Doxo vs. Doxo-NMN and *p* < 0.0001 Ctrl vs. Doxo-NMN). These results reflect a protective effect of NMN treatment on Doxo-induced alterations in cardiac contractility. Accordingly, Doxo injection resulted in 3- and 5-fold increases in LDH and troponin-I releases, respectively when compared to the Ctrl group (*p* < 0.0001 for both LDH and troponin-I). NMN Treatment significantly attenuated the Doxo-induced plasma LDH and troponin-I level decreases (35% and 34% reduction, respectively) (LDH: 2031.6 ± 241.8 U/L (Doxo) vs. 1321.0 ± 102.0 U/L (Doxo-NMN), *p* < 0.001 and troponin-I: 560.0 ± 101.5 pg/mL (Doxo) vs. 369.6 ± 40.8 pg/mL (Doxo-NMN), *p* < 0.05), but they remained significantly higher than those measured in Ctrl mice (LDH: 705.9 ± 70.5 U/L (Ctrl), *p* < 0.05 and troponin-I: 112.5 ± 14.7 pg/mL (Ctrl), *p* < 0.01) (Figure 1E). These results demonstrate the cardioprotective effect of NMN during treatment with Doxo.

### 3.2. NMN Affects the NAD+ Metabolome in Mice Receiving Acute Doxo Treatment

To evaluate the in vivo impact of Doxo and NMN treatments on NAD+ metabolome, LC-MS analyses were performed on whole blood and heart tissues collected from Ctrl, Doxo and Doxo-NMN mice at D5 (Figure 1F,G). Among the different NAD+ metabolites evaluated, blood levels of NAD+ and NMN are significantly reduced after Doxo treatment compared with the Ctrl group (NAD+: −19%, *p* < 0.01 and NMN: −47%, *p* < 0.0001) (Figure 1F). Treatment with NMN did not prevent these decreases, but induced important increases in circulating levels of NAM, n-MetNAM and Me2Py compared to both Ctrl and Doxo groups (Doxo-NMN vs. Ctrl: 6-fold increase NAM (*p* < 0.0001), 11-fold increase n-MetNAM (*p* < 0.0001) and 12-fold increase Me2Py (*p* < 0.0001); Doxo-NMN vs. Doxo: 8-fold increase NAM (*p* < 0.0001), 7-fold increase n-MetNAM (*p* < 0.001) and 6-fold increase Me2Py (*p* < 0.01)) (Figure 1F). In the heart, Doxo did not induce any significant changes in the tissue levels of NAD+ and its metabolites compared to those measured in hearts from the Ctrl group. However, trends towards diminished levels of NAD+, NMN and NAAD were noticed (Figure 1G). While NMN treatment had no impact on NMN and NAM contents in Doxo-treated hearts, it significantly increased the NAD+, NAAD, n-MetNAM and Me2Py levels compared to the Ctrl and Doxo groups (NAD+: 1.2-fold increase (*p* < 0.05 vs. Ctrl) and 1.5-fold increase (*p* < 0.0001 vs. Doxo); NAAD: 1.3-fold increase (*p* < 0.05 vs. Ctrl) and 1.9-fold increase (*p* < 0.0001 vs. Doxo); n-MetNAM: 12-fold increase (*p* < 0.001 vs. Ctrl) and 12-fold increase (*p* < 0.0001 vs. Doxo) and Me2Py: 14-fold increase (*p* < 0.0001 vs. Ctrl) and 10-fold increase (*p* < 0.0001 vs. Doxo)) (Figure 1G). These data show that acute treatment with Doxo significantly alters the NAD+ metabolome at the circulating level and to a lesser extent in the heart tissue. Additionally, we found that NMN administered by i.p. injection is bioavailable and metabolized in the heart tissues.

### 3.3. Treatment with NMN Reduces the Expression of Genes Associated with Mitochondrial Damages, Oxidative Stress, Inflammation, and Apoptosis Induced by the Acute Doxo Treatment

**Figure 2:**
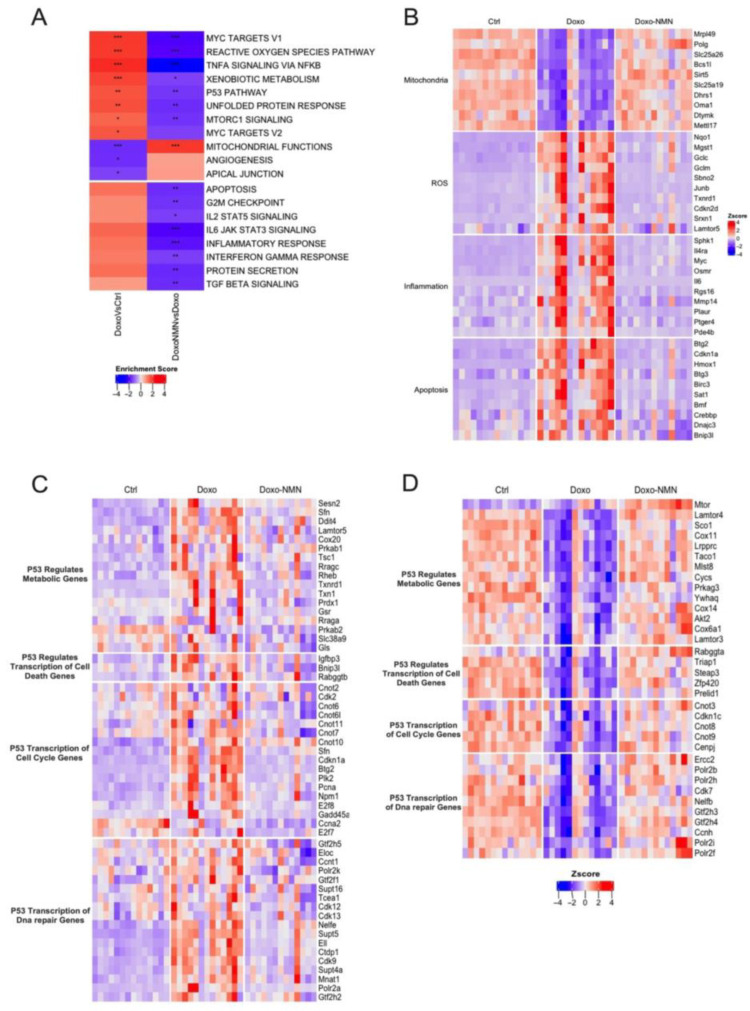
Treatment of NMN prevents the cardiac transcriptional changes induced by the acute Doxo treatment. (**A**) Heatmap of canonical pathways enriched in hearts. Normalized enrichment score reflects the up- (Enrichment score > 0; red colors) and down- (Enrichment score < 0, blue colors) expressed pathways. * *p* < 0.05; ** *p* < 0.01; *** *p* < 0.001. (**B**–**D**) Heatmap of gene expression related to apoptosis, inflammation, mitochondria, Ros (**B**), p53 signaling (up-regulated (**C**) or down-regulated (**D**) by Doxo vs. Ctrl). Red colors indicate Z score > 0 (up-regulation) and blue colors indicate Z score < 0 (down-regulation). Genes in panel B were selected from the corresponding Hallmark terms and genes in panels C and D were selected from the corresponding Reactome terms.

To decipher the molecular mechanisms involved in Doxo cardiotoxicity and those supporting the observed protective effects of NMN treatment, we performed RNA-seq analysis on heart tissues collected 5 days after Doxo administration, in Ctrl, Doxo and Doxo-NMN groups (Figure 2). Pairwise comparison between Doxo vs. Ctrl mice and Doxo-NMN vs. Doxo mice identified 3874 and 2207 differentially expressed genes (adjusted *p*-value < 0.05 and FC cutoff of 1.5), respectively. Interestingly, among the genes dysregulated by Doxo injection, NMN treatment maintained the expression levels of 1628 genes comparable to those observed in hearts of Ctrl mice, i.e., 42% of the Doxo modulated genes. Canonical pathways enrichment (Hallmark pathways from MSigDB collections) yielded eight significantly up-regulated and three significantly down-regulated pathways in hearts of the Doxo group compared to the Ctrl group (Figure 2A). Interestingly, amongst these pathways, seven were down-regulated and one up-regulated significantly in the presence of NMN treatment. Specifically, NMN administration significantly down-regulated genes involved in pathways of metabolism, cell proliferation, apoptosis (MTORC1 signaling; P53 pathway, Myc targets v1), cellular stress response (unfolded protein response, reactive oxygen species pathway), detoxification (xenobiotic metabolism) and the immuno-inflammatory (TNFα signaling via NF-κB). Additionally, the presence of NMN led to significant up-regulation of the expressions of genes involved in mitochondrial function associated pathways. Interestingly, we also observed that the presence of NMN had a down-regulatory effect on 8 pathways not significantly impacted by Doxo, in particular, inflammation and cytokine response pathways (Il2/Stat5, Il6 jak/stat3, TGF-β signaling Interferon γ response), as well as apoptosis, cell proliferation (G2M checkpoint) and protein secretion pathways.

More precisely, hearts from Doxo-treated mice exhibited decreased expression in 283 genes associated with mitochondrial integrity and functions in comparison to the Ctrl mice. Among these, NMN administration prevented the modification of the expression of 232 genes including in the top 10 with the highest *p*-value, genes related to mitochondrial protein translation (*Mrpl49* and *Mettl17*), mitochondrial DNA replication (*Polg* and *Dtymk*), mitochondrial transporters (*Slc25a19* and *Slc25a26*), mitochondrial respiratory chain (*Bcs1l*), mitochondrial energy metabolism (*Sirt5*, *Dhrs1* and *Oma1*) (Figure 2B). Moreover, Doxo exposure also resulted in major alteration of genes involved in ROS production and elimination. Twenty-two genes were increased following the Doxo injection and NMN treatment-maintained expression levels of 20 of them comparable to those of Ctrl hearts. Our RNA-seq analysis showed that the most significantly impacted gene by NMN were associated with the main enzymatic branches of the antioxidant network, such as glutathione transferase (*Mgst1*), Glutamate-Cysteine Ligase (*Gclc* and *Gclm*), but also redox proteins such as thioredoxins (*Txnrd1*), sulfiredoxin (*Srxn1*) and xenobiotic detoxification (*Nqo1*), oxidative stress regulator (*Sbno2*, *Junb* and *Lamtor5*), pro-oxidative agent (*Cdkn2d*) (Figure 2B). Naturally, along with genes related to mitochondrial function and ROS production/removal, 56 genes associated with the inflammatory response were also up-regulated after Doxo injection and expression of 51 of these genes were down-regulated by NMN. These genes belong to the production of cytokines and chemokines (*Il6*, *IL4rs*, *Osmr* and *Rgs16*), the modulation of their activity (*Myc*, *Mmp14*, *Sphks*, *Pde4b* and *Ptger4*), as well as to the immune cell infiltration (*Plaur*) (Figure 2B). Ultimately, disturbance in mitochondrial function, ROS damage and inflammation, lead to apoptosis. Consistently, NMN treatment prevented the increased expression of most apoptotic genes induced by Doxo injection (i.e., 45 of the 51 genes modulated by Doxo). These NMN-impacted genes encode proteins involved in initiation (*Btg2*, *Btg3*, *Bmf* and *Bnip3l*) and regulation (*Birc3*, *Dnajc3*, *Crebbp* and *Cdkn1a*) of intrinsic apoptosis signaling and ferroptosis (*Sat1* and *Hmox1*). Overall, these data suggest that at the cardiac level, NMN largely prevents Doxo-induced deleterious alterations in gene expression.

### 3.4. NMN Administration Is a Key Modulator of p53 Pathway Transcriptomic Changes Induced by Doxo Acute Treatment

Pathway analysis revealed that, in the context of cardiotoxicity induced by acute Doxo treatment, NMN appears to be an important down regulator of the activated p53-associated gene network that orchestrate the cellular response and cell cycle arrest, senescence and apoptosis [33,34,35]. Analysis of the p53-associated genes present in the Reactome database, revealed that 201 transcripts were impacted by acute Doxo treatment (65% of the genes in the Reactome database associated with p53). Remarkably, the presence of NMN significantly attenuated the variation of 171 of these genes representing 85% of the Doxo impacted genes (Figure 2C,D). As described in Figure 2C,D, all biological functions controlled by p53 are targeted by the protective effect of NMN during acute Doxo treatment, i.e., regulation of metabolic genes (17 genes down- and 14 up-regulated), cell death gene transcription (3 genes down- and 5 up-regulated), cell cycle gene transcription (17 genes down-regulated and 5 down-regulated), DNA repair genes (18 genes down- and 10 up-regulated). In addition, NMN prevents Doxo-induced expression changes of 82 genes (30 up- and 52 down-regulated) involved in the regulation of p53 activity (Supplementary Figure S1).

### 3.5. Orally Administered NMN Is Bioavailable and Improves Survival and Bodyweight Loss Induced by Doxo

**Figure 3:**
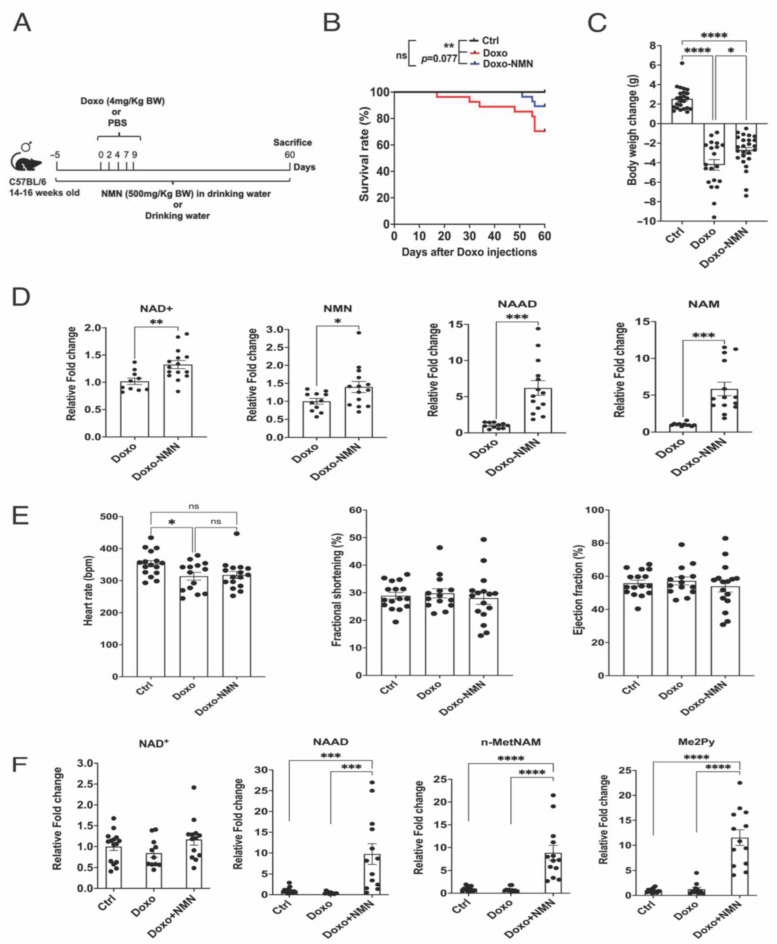
Orally administered NMN is bioavailable and improves survival and body weight loss impaired by a chronic Doxo regime. (**A**) Study design for the chronic regimen in Doxo-induced cardiotoxicity mouse model. Preventive treatment with NMN (500 mg/kg/day, po) was initiated 5 days before five injections of Doxo (4 mg/kg/day, i.p., cumulative 20 mg/kg) and continued over 60 days. (**B**) Survival curve (percentage, %) of ctrl, Doxo and Doxo-NMN mice. Log-rank (Mantel–Cox) test. ** *p* < 0.01, ns = not significant. (**C**) Body weight change (grams, g) at the end of the treatment period. Body weight change has been calculated as follows: Body weight at the day of sacrifice (day 60)—body weight at the day of Doxo-injection (day 0). One-way Anova followed by Tukey’s post-test. * *p* < 0.05, **** *p* < 0.0001. (**D**) Levels of NAD+, NMN, NAAD and NAM (relative to Doxo group) in blood measured by LC-MS/MS. One-way Anova followed by Tukey’s post-test. * *p* < 0.05, ** *p* < 0.01, *** *p* < 0.001. (**E**) Heart rate, ejection fraction and fraction shortening were assessed by 2D-echocardiography on day 53 after the first Doxo injection. One-way Anova followed by Tukey’s post-test. * *p* < 0.05, ns = not significant. (**F**) Levels of NAD+, NAAD, n-MetNAM and Me2Py (relative to Ctrl group) in heart tissue measured by LC-MS/MS. One-way Anova followed by Tukey’s post-test. *** *p* < 0.001, **** *p* < 0.0001. Data are shown as means ± the standard error.

To evaluate whether the oral NMN administration could prevent the adverse effects of a chronic Doxo treatment, male C57BL/6 mice at 14–16 weeks of age were injected intraperitoneally with PBS (vehicle) or with 4 mg/kg/day of Doxo for five times for a cumulative dose of 20 mg/kg. Doxo-treated mice either received regular drinking water (Doxo) or 500 mg/kg/day NMN in their drinking water (Doxo-NMN), whereas control mice (Ctrl) received regular drinking water for the entire duration of the study (Figure 3A). This regimen of Doxo was chosen based on previous studies showing the increase in skeletal muscles’ cellular senescence [26] and weakness [36], whereas the dosage of NMN used in this study was previously proven to be safe and effective in increasing NAD+ levels in peripheral tissue [37,38,39,40,41]. Survival rates were compared between groups up to 60 days after the first Doxo injection using the Kaplan–Meier test. Doxorubicin-treated mice had a survival rate of 70.4% that was increased to 89.3% by NMN (*p* = 0.077) (Figure 3B). In agreement with prior studies [42,43], Doxo induced a severe and progressive decrease in body weight that was attenuated by NMN (Supplementary Figure S2A). Indeed, the Doxo-induced body weight loss was significantly mitigated by NMN at the end of the study (−2.79 ± 0.34 g (Doxo-NMN) vs. −4.24 ± 0.54 g (Doxo), *p* = 0.024) (Figure 3C). Based on body composition analysis, the percentages of lean mass were comparable between the different groups whereas the percentage of fat mass was dramatically decreased in the Doxo group compared to the Ctrl group at 50 days after the first Doxo injection and consistently with the body weight results, mice treated with NMN showed a trend toward rescue in fat mass (Supplementary Figure S2B). To confirm the bioavailability of NMN by oral administration, we performed targeted, LC-MS-based metabolomics for the semi-quantitative measurement of NAD+ -related metabolites in whole blood collected from Doxo and Doxo-NMN mice at the end of the study. Indeed, the oral administration of NMN in drinking water was able to significantly increase the circulating levels of NAD+, NMN, nicotinamide (NAM) and nicotinic acid adenine dinucleotide (NAAD) that is considered a highly sensitive biomarker of increased NAD+ metabolism [44] (Figure 3D).

### 3.6. The Doxo-Induced Alteration in the Expression of Genes Related to Mitochondrial Functions, DNA Damage and Apoptosis Was Improved by the Oral NMN Treatment in the Heart

**Figure 4:**
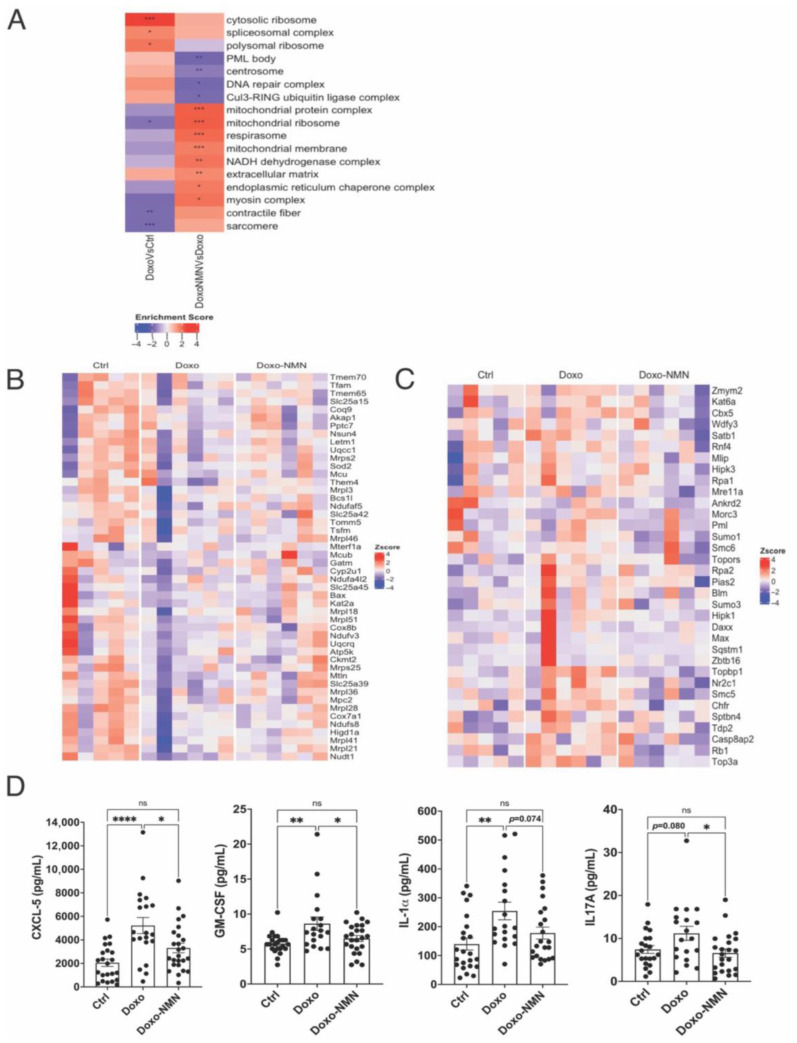
Transcriptomic signature of hearts and plasma cytokines and chemokines analysis in the chronic Doxo treatment. (**A**) Heatmap of enriched GO terms in hearts. Normalized enrichment score reflects the up- (Enrichment score > 0) and down- (Enrichment score < 0) adjusted *p*-values are shown superimposed on the color gradient * *p* < 0.05; ** *p* < 0.01; *** *p* < 0.001. Heatmap of genes related to mitochondrial functions (**B**) and PML-NB (promyelocytic leukemia- nuclear bodies). (**C**) in hearts. Red colors indicate Z-score > 0 (up-regulation); blue colors indicate Z-score < 0 (down-regulation); white color indicates Z-score = 0 (no difference). For all data n = 5–6 for each group. (**D**) Plasma levels (picograms, pg; milliliter, mL) of C-X-C motif chemokine 5 (CXCL5), Granulocyte-macrophage colony-stimulating factor (GM-CSF), Interleukin-1α (IL-1α) and Interleukin 17A (IL17A) quantified by Mouse Cytokine/Chemokine Array 18-plex assay at 59 days after the first Doxo injection. One-way Anova followed by Tukey’s post-test. * *p* < 0.05, ** *p* < 0.01, **** *p* < 0.0001, ns = not significant. Data are shown as means ± the standard error.

Considering that there is not a common protocol to induce cardiotoxicity by chronic Doxo administration in the literature [45] and having demonstrated the cardioprotective effects of NMN treatment after an acute Doxo regime (Figure 1), we aimed to investigate the effects of NMN on cardiac functionality using the chronic Doxo treatment adopted in this study. As depicted in Figure 3E, at 53 days after the first Doxo injection, despite a significant decline in heart rate in Doxo-mice, we could not detect any significant changes in fractional shortening (FS) and ejection fraction (EF) in Doxo-mice by 2D echocardiography analysis, suggesting a delayed development of cardiac defects. To be noted, heart rate, FS and EF were comparable between the different groups at 30 days after the first Doxo injection (data not shown). Nevertheless, the oral NMN treatment was able to significantly increase the intracellular contents of NAAD, n-MetNAM and Me2Py, metabolites involved in the NAD+ turnover, in heart tissue collected at the end of the study (Figure 3F and Figure S2C). Therefore, we performed RNA-seq analysis to evaluate the underlying molecular changes at an early stage of Dox-induced cardiac impairment. We identified nine genes differentially expressed in Doxo vs. Ctrl and none in Doxo-NMN vs. Doxo (*p*.adj < 0.05). Despite the small number of differentially expressed genes, the GSEA analysis on GO terms identified 11 terms up-regulated and 15 down-regulated in the heart of Doxo mice compared to Ctrl mice whereas 34 GO terms were up-regulated and 15 down-regulated in the heart of Doxo-NMN group compared to Doxo group. Interestingly, pathways related to mitochondrial functions and promyelocytic leukemia-nuclear bodies (PML-NB) were consistently modulated by NMN in both the acute and chronic regimen of Doxo (Figure 2B–D and Figure 4A). Specifically, in the mitochondrial functions pathway, the chronic Doxo-induced reduction in the expression of genes related to the mitochondrial transcription (*Tfam*, *Tsfm*, *Mterf1a*), mitochondrial proteins translation (*Mrps2*, *Mrpl18*, *Mrpl21*, *Mrps25*, *Mrpl28*, *Mrpl36*, *Mrpl41*, *MrpI46*, *Mrpl51*, *Nsun4*), mitochondrial transporters (*Mpc2*, *Slc25a42*, *Slc25a45*, *slc25a39*, *Slc25a15*, *Mcu*), antioxidant defense (*Sod2*, *Slc25a39*, *Nudt1*) and the respirasome complexes (*Ndufv3*, *Ndufaf5*, *Ndufs8*, *Ndufa 4I2*, *Uqcc1*, *Coq9*, *Uqcrq*, *Cox8b*, *Cox7a1*, *Atp5k*) were mitigated by NMN treatment (Figure 4B). Conversely, in the PML-NB pathway, Doxo increased the expression of genes involved in the DNA damage and repair (*Rpa2*, *Tdp2*, *Smc5*, *Rpa1*, *Mre11a*, *Topbp1*, *Blm*, *Smc6*, *Topors*, *Rb1*, *Top3a*) and apoptotic process (*Daxx*, *Hipk3*, *Hipk1*, *Zbtb16*, *Pias2*, *Casp8ap2*, *Pml*, *Max*, *Sqstm1*) that were down-regulated by the NMN treatment (Figure 4C).

Doxo treatment is known to cause systemic inflammation [46,47]. Compared to the acute study, we did not detect any significant changes in the expression of genes associated with inflammatory response in the hearts derived from the chronic study. Nevertheless, the blood plasma levels of the proinflammatory cytokines and chemokines, GM-CSF, IL-1α, CXCL5 and IL17A were increased in Doxo group and normalized by NMN (GM-CSF: 8.63 ± 0.95 pg/mL (Doxo) vs. 6.51 ± 0.43 pg/mL (Doxo-NMN), *p* = 0.034; IL-1α:254.4 ± 30.6 pg/mL (Doxo) vs. 177.7 ± 20.7 pg/mL (doxo-NMN), *p* = 0.074; CXCL5: 5218 ± 684 pg/mL (Doxo) vs. 3308 ± 407 pg/mL (Doxo-NMN) *p* = 0.017; IL17A: 11.18 ± 1.64 pg/mL (Doxo) vs. 6.59 ± 0.97 pg/mL (Doxo-NMN) *p* = 0.021) (Figure 4D) whereas IL-6, TNFα, IL1b, IL2, IL4, IL5, IL10, IL13, CXCL-1, CXCL2, CCL2, INF-g, IL-7 and IL-12p70 levels were similar among the different groups or undetectable (data not shown). Increased levels of IL-1α, IL17A and CXCL5 have been linked to sarcopenia and cardiotoxicity [48,49,50].

### 3.7. The Doxo-Induced Loss of Physical Activity Is Prevented by Oral NMN Administration

**Figure 5:**
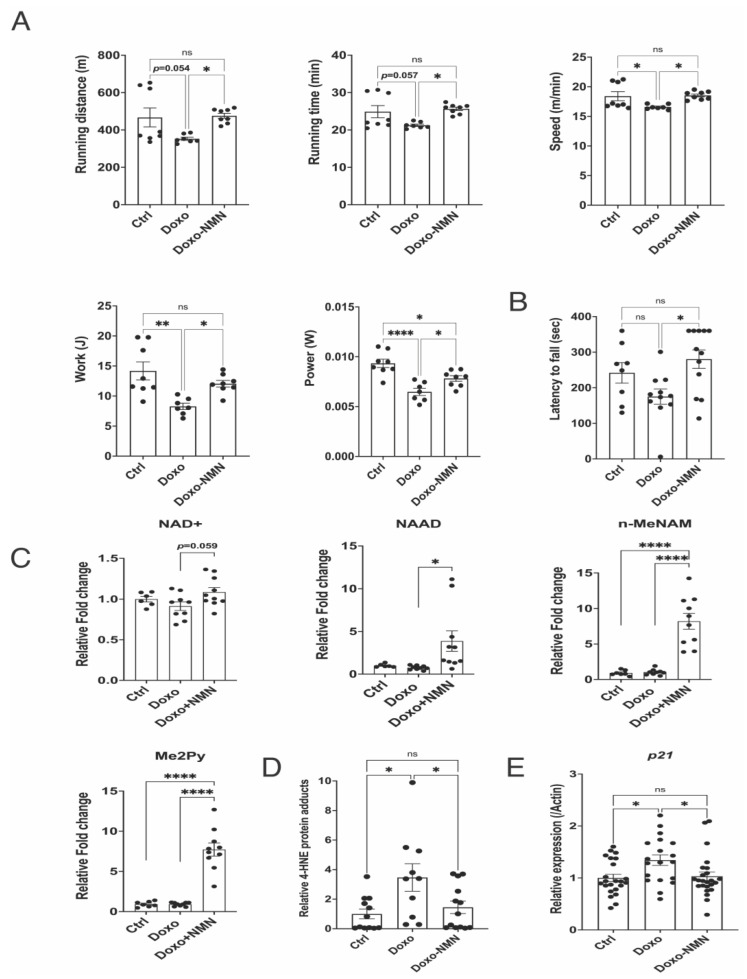
Oral NMN administration increases NAD+ metabolism in skeletal muscle and prevents Dox-induced physical impairment. (**A**) Uphill treadmill exhaustion test performed at 45 days after the first Doxo injection. Graphs show running distance (meters, m), running time (minutes, min), maximal speed (meters/minutes, m/min), work (Joule, J) and Power (Watt, W). Work and Power were calculated as described in Methods. One-way Anova followed by Tukey’s post-test. * *p* < 0.05, ** *p* < 0.01, **** *p* < 0.0001, ns = not significant. (**B**) Rotarod test performed 45 days after the first Doxo injection. Graph shows the latency to fall from a motorized rotarod device (seconds, sec). One-way Anova followed by Tukey’s post-test. * *p* < 0.05, ns = not significant. (**C**) Levels of NAD+, NAAD, n-MetNAM and Me2Py (relative to Ctrl group) in gastrocnemius tissue measured by LC-MS/MS. One-way Anova followed by Tukey’s post-test. * *p* < 0.05, **** *p* < 0.0001. (**D**) Levels of 4-HNE protein adducts (relative to Ctrl group) in gastrocnemius tissue. One-way Anova followed by Tukey’s post-test. * *p* < 0.05, ns = not significant. (**E**) P21 levels in gastrocnemius muscle. mRNA levels were quantified by RT-qPCR and normalized to actin mRNA levels. The average value of Ctrl was set at 1. One-way Anova followed by Tukey’s post-test. * *p* < 0.05, ns = not significant. Data are shown as means ± the standard error.

Doxorubicin treatment is associated with muscle weakness and fatigue [51,52]. To evaluate whether NMN administration could potentially ameliorate the Doxo-induced reduction in physical activity, we performed an uphill treadmill exhaustion test on Ctrl, Doxo and Doxo-NMN mice to assess their exercise capacity at 45 days after the first Doxo injection. For all the parameters analyzed, the Doxo-NMN group performed as efficiently as the control group. Indeed, Doxo-NMN mice ran significantly longer compared to the Doxo group (25.65 ± 0.46 min (Doxo-NMN) vs. 21.26 ± 0.31 min (Doxo), *p* = 0.019). Since Doxo-NMN mice could keep running at higher speed compared to Doxo (18.53 ± 0.25 m/min (Doxo-NMN) vs. 16.69 ± 0.16 m/min (Doxo), *p* = 0.032), the difference in running distance was 35% higher in Doxo-NMN mice compared to Doxo group (475.9 ± 13.4 m (Doxo-NMN) vs. 353.0 ± 8.5 m (Doxo), *p* = 0.036). Accordingly, work and power generated by Doxo-NMN were 45.6% and 20.9% higher compared to Doxo group, respectively (Work: 12.06 ± 0.55 joule (Doxo-NMN) vs. 8.28 ± 0.52 joule (Doxo), *p* = 0.040, Power: 0.00782 ± 0.00028-watt (Doxo-NMN) vs. 0.00648 ± 0.00036 watt (Doxo), *p* = 0.043) (Figure 5A). In line with the treadmill results, strength and motor coordination, measured as latency to fall off an accelerating rotarod device, were significantly improved in the Doxo-NMN group compared to Doxo (280.4 ± 25.9 s (Doxo-NMN) vs. 175.2 ± 21.4 s (Doxo), *p* = 0.012) (Figure 5B). Gastrocnemius muscle tissue was collected from Ctrl, Doxo and Doxo-NMN mice at the end of the study to assess the effect of NMN treatment on skeletal muscle NMN-derived metabolites. Although muscle NAD+ content was mildly enhanced by NMN, the levels of NMN, NAM, N-methyl-nicotinamide (n-MetNAM), methyl-2-pyridone-5-carboxamide (Me2Py) were significantly increased suggesting that NMN treatment greatly enhanced muscle NAD+ turnover (Figure 5C and Figure S2D).

Oxidative stress due to an increased production of reactive oxygen species (ROS) has been proposed as one of the causes of Doxo-induced skeletal muscle dysfunction [36]. As a consequence of oxidative stress-induced lipid peroxidation, the production of biologically active aldehydes, such as 4-hydroxy-2-nonenal (4-HNE) is increased and the formation of 4-HNE-adducts with muscle proteins has been shown to induce oxidative damage after Doxo treatment [53,54]. Remarkably, our results show that the Doxo-induced increase in 4-HNE protein adducts was significantly reduced in gastrocnemius muscle by NMN treatment at day 60 from the first Doxo injection (3.47 ± 0.93-fold increase (Doxo) vs. 1.45 ± 0.42-fold increase (Doxo-NMN), *p* = 0.048) (Figure 5D). Lastly, to evaluate the possible increase in senescence cells within muscle after Doxo treatment, we performed RT-qPCR analysis of senescent marker genes (p16 and p21) in gastrocnemius muscle. Consistent with previous results using the same regimen of Doxo administration [26], p21 mRNA levels were significantly increased by Doxo treatment in gastrocnemius muscle; whereas the expression of p16 was barely detectable at day 60 from the first Doxo injection (data not shown). Interestingly, NMN treatment was able to effectively revert the Doxo-induced overexpression of p21 (1.34 ± 0.10-fold increase (Doxo) vs. 1.03 ± 0.08-fold increase (Doxo-NMN), *p* = 0.034) (Figure 5E). These results are also in line with the reduced expression of p21 (*Cdkn1a*) by NMN in heart tissue from the acute study (Figure 2B).

All together these data indicate that the oral NMN administration is sufficient to increase the skeletal muscle NAD+ metabolism and consequently ameliorate the Doxo-induced impairment in physical capacity possibly by reducing oxidative stress and cellular senescence in skeletal muscle.

## 4. Discussion

Cardiac and skeletal muscle dysfunctions induced by anthracycline-based chemotherapy is known to reduce quality of life and increase the risk of death from cancer survivors [55,56]. In particular, the clinical use of Doxo is associated with severe and irreversible cardiotoxicity [57,58] and skeletal muscle impairment [59,60,61]. Doxo-induced cardiotoxicity can be detected within days, months, or years after chemotherapy, even after cancer remission [62]. Unfortunately, there are no effective strategies to prevent or treat the side effects of Doxo-induced toxicity.

Our aim was to investigate, in the context of acute and chronic Doxo administration, the possible protective effects of NMN against cardiotoxicity and skeletal muscle weakness which is characterized by diminished exercise capacity and increased fatigue.

In the acute toxicity setting (Figure 1A), Doxo induced a significant reduction in body weight and elevated mortality of animals (52%), which is thought to be primarily due to heart failure as described previously [63,64] although damage to other organs such as the liver and lungs were also observed [64,65]. Accordingly, we found that injection of Doxo resulted in marked impairment of cardiac functions, evidenced by significant decreases in HR, EF and FS. This was further confirmed by the concomitant increase in the plasma levels of systemic markers of heart distress (LDH and troponin-I) and profound alterations in the transcriptome of cardiac tissue. Remarkably, the intraperitoneal administration of NMN allowed its bioavailability and metabolism in heart tissue (Figure 1G) and prevented Doxo-induced death (Figure 1B), reduced body weight loss (Figure 1C) and preserved the cardiac functions (Figure 1D,E). Transcriptomic analysis suggests that NMN confers protection to cardiac cells by preserving mitochondrial integrity and function, reducing oxidative stress and inflammatory response, ultimately leading to limitation of apoptosis. However, to further confirmed these beneficial effects at the functional level, additional experimental work in required. Overall, these results indicate promising cardioprotective effects of NMN against Doxo toxicity. These findings are consistent with previous work suggesting that NMN could protect cardiomyocytes from Doxo-induced toxicity and further support the cardioprotective potential of NMN already demonstrated in various cardiac conditions such as heart failure, cardiac fibrosis and cardiotoxicity [24,63,66,67,68].

Mechanistically, the protective effect of NMN in Doxo-induced cardiotoxicity could be mediated by activation of SIRT1. Indeed, the enhancement of intracellular NAD+ levels increases the activity of SIRT1, an NAD+-dependent enzyme, which can protect against oxidative damage [69,70], restore mitochondrial biogenesis and cell cycle arrest and suppress apoptotic mechanisms [71,72,73]. Moreover, the protection against cardiotoxicity provided by nicotinamide riboside (NR), another NAD+ biosynthetic precursor, is dependent on SIRT1 activity since its chemical inhibition prevents its cardioprotective effects [74]. However, more detailed data on the mechanisms of NAD+/SIRTs regulation are needed, especially to identify the involvement of other NAD+ consuming enzymes, such as PARPs.

Furthermore, in the context of cardiotoxicity induced by acute Doxo treatment, pathway analysis of the transcriptome clearly revealed the dysregulation of gene expressions related to the p53 pathway coordinating the cellular response and cell cycle arrest as well as senescence and apoptosis [33,34,35]. Acute Doxo treatment induced dysregulation in both p53 target genes, including cell cycle, DNA damage and apoptosis (Figure 2C) and expression of p53 pathway modulator genes (Supplementary Figure S1). Remarkably, NMN treatment restores all these genes to basal levels. These results are consistent with previous data indicating that acute Doxo-induced cardiac dysfunction, cardiomyocyte apoptosis and cardiac mass reduction occur via a p53-dependent pathway [75] and with studies showing that NR and niacinamide affect p53-regulated cellular responses [74,76]. Mechanistically, NMN could act directly on p53. Indeed, it has been shown that NAD+ was able to bind to p53 tetramers and alter p53 DNA binding specificity thereby modulating the activation of a subset of p53 transcriptional targets [76]. Furthermore, by boosting NAD+ in heart cells, NMN could also promote de-acetylation of p53 via the NAD+/SIRT1 signaling pathway [74]. Collectively, these data indicate that Doxo-induced acute cardiac dysfunction and cardiomyocyte apoptosis may occur via p53-dependent pathways, which can be dampened by NMN administration.

In our chronic study, Doxo treatment reduced the survival rate by 30% and induced a severe decrease in body weight 60 days after the first Doxo injection (Figure 3A,B). The rapid loss of adipose mass induced by Doxo has been previously ascribed to an increase in lipolysis [77] and a concomitant decrease in adipogenesis and lipogenesis [78]. Despite a significant decline in heart rate, we did not detect any significant changes in EF or FS at 53 days (Figure 3E) as well as at 30 days post Doxo treatment (data not shown), suggesting that this regimen of Doxo could potentially induce more pronounced cardiac defects at a later time point or in different experimental models. Indeed, in Wistar rats, chronic injections of Doxo at cumulative doses of 12 and 15 mg/kg resulted in significant cardiac tissue damage that was significantly attenuated by NMN [68] and even totally prevented by the combination of NMN with troxerutin (vitamin P4) [24]. On the other hand, our data show that the physical capacity measured by the treadmill exhaustion and rotarod tests was significantly reduced by Doxo (Figure 5), indicating skeletal muscle weakness. Remarkably, the administration of NMN in the drinking water was able to greatly ameliorate the survival rate (Figure 3B), the body weight loss (Figure 3C) and the physical capacity (Figure 5A,B) impaired by Doxo. Moreover, we showed that the oral supplementation of NMN is efficient in enhancing the levels of NAD+ and NAD-related metabolites in the bloodstream (Figure 3D) as well as in peripheral tissue such as the heart (Figure 3F) and gastrocnemius muscle (Figure 5C and Figure S2D).

In line with previous studies, the short-term and long-term NMN supplementation has proven to increase locomotor functions and muscle strength in aged mice [79,80] as well as in healthy human subjects [81,82]. However, to the best of our knowledge, the results presented here are the first to demonstrate that the oral NMN administration preserves the exercise capacity in a context of chemotherapeutic regimen. Interestingly, a recent study has shown that the intravenous administration of another biosynthetic NAD+ precursor, NR (300 mg/kg) in Wistar rats subjected to a chronic Doxo treatment (10 mg/kg cumulative dose) has little to no effect on exercise capacity at 30- and 60 days post Doxo administration [83]. Therefore, additional studies are needed to compare different NAD+ biosynthetic precursors in the same experimental conditions and animal models in order to identify the most efficient NAD+-boosting strategy to counteract Doxo-induced side-effects.

Nevertheless, it is important to mention that compared to previous reports [24,68,74,83,84] using biosynthetic precursors, such as NMN, NR and Nicotinic acid riboside (NAR) in animal models of Doxo-induced toxicity, we demonstrated that NMN supplementation is sufficient to improve the physical capacity impaired by Doxo using a clinically relevant route of administration and a concentration (500 mg/kg/day) that is below the recently reported NOAEL (No-Observable Adverse Effect Level) for NMN in Sprague Dawley rats [37].

Mechanistically, our chronic study reveals that the oral NMN administration was able to attenuate the Doxo-induced increase in circulating proinflammatory cytokines and chemokines (Figure 4D), to enhance the cardiac expression of genes involved in mitochondrial biogenesis and oxidative metabolism (Figure 4B) and concomitantly mitigate the overexpression of genes related to DNA damage and apoptosis (Figure 4C).

At the skeletal muscle level, NMN reverted the accumulation of 4-HNE protein conjugates (Figure 5D), a biomarker of oxidative stress-induced lipid peroxidation [85]. The variety of different cellular pathways impacted by NMN is not surprising considering the pleiotropic functions that NAD+ has on energy metabolism as redox cofactor and on DNA repair, cellular growth and survival as substrate of enzymes such as SIRTs and poly (ADP-ribose) polymerases [15].

Lastly, Doxo administration has been proven to induce cellular senescence in mouse tissues such as heart [13] and gastrocnemius muscle [26]. Senescence is mediated via activation of either one or both p53/p21 and p16/RB (Retinoblastoma) pathways in which the up-regulation of p21 and p16 play a key role in the induction and persistence of senescence, respectively [86,87]. Interestingly, NMN administration was able to significantly reduce the expression of p21 (*Cdkn1a*) in gastrocnemius muscle (Figure 5C) as well as in heart (Figure 2B). Little is known about the role of NAD+ metabolism during cellular senescence, therefore it would be interesting to evaluate the potential effects of NMN supplementation on senescence and SASP (senescence-associated secretory phenotype).

## 5. Conclusions

In summary, we demonstrated that NMN reverses the Doxo-induced mortality and cardiotoxicity by implementing two regimens of Doxo, acute and chronic. Moreover, we showed for the first time that the oral NMN administration prevents the impairment in physical capacity that along with the onset of cardiac dysfunctions are the most common side-effects provoked by chemotherapy.

At the molecular level, NMN protects from the Doxo-induced transcriptomic alterations in pathways related to mitochondrial functions, apoptosis, oxidative stress and inflammation in heart tissue. In particular, we observed that the cellular pathways mediated by p53, which is a master regulator of cellular response to insults, cell cycle arrest, senescence and apoptosis, are conserved from Doxo-driven hyper activation in the heart. In conclusion, given its positive toxicology and safety profile, NMN might represent a promising prophylactic strategy to prevent deleterious toxic side effects of chemotherapies.

## Data Availability

RNA-seq data were deposit in GEO database (reference number: GSE221003).

## Supplementary Materials

The following supporting information can be downloaded at: https://www.mdpi.com/article/10.3390/cells12010108/s1, Table S1 (cardiac function in acute toxicity study): The effect of NMN treatment on cardiac dysfunction induced by acute toxicity of Doxo; Figure S1: Effect of NMN treatment on regulation of p53 activity in the acute Doxo study; Figure S2: The effects of NMN administration on Doxo-induced body weight decrease and NAD-related metabolites in heart and gastrocnemius muscle tissue.
