# Supplementary material for: Nicotinamide Mononucleotide Administration Prevents Doxorubicin-Induced Cardiotoxicity and Loss in Physical Activity in Mice
Source: Cells. 2022 Dec 27;12(1):108. doi: 10.3390/cells12010108 (PMC9818647; doi:10.3390/cells12010108)
Supplement: Supplementary file 1 [file cells-12-00108-s001.zip › cells-2085354-supplementary.pdf]

Table S1: The effect of NMN treatment on cardiac dysfunction induced by acute toxicity of Doxo.
Left ventricular volume and internal diameter, interventricular septum thickness and left ventricular posterior wall were assessed by echocardiography on day 5. Mice were then euthanized and hearts and tibiae were collected, weighed or measured. Data are shown as means ± the standard error.Nonparametric Kruskal-Walli's test followed by Dunn's post-test or One-way Anova followed by Tukey's post-test. *p < 0.05, **p < 0.01, ***p < 0.001, ****p < 0.0001, ns = not significant. LV Volume: Left ventricular volume; LVID: Left ventricular internal diameter; IVS: Interventricular septum thickness; LVPW: Left ventricular posterior wall; d: diastole; s: systole.

Parameter	Ctrl		Doxo		Doxo-NMN		Summary Ctrl vs. Doxo	Summary Ctrl vs. Doxo-NMN	Summary Doxo vs. Doxo-NMN
	Mean	SE	Mean	SE	Mean	SE			
Heart weight / tibia length (mg/mm)	7.67	0.11	5.79	0.11	6.06	0.10	****	****	ns
LV Volume; d (µL)	22.71	0.45	18.27	1.14	21.02	0.63	***	ns	ns
LV Volume; s (µL)	7.28	0.18	10.92	0.98	8.95	0.30	****	***	ns
LVID; d (mm)	3.80	0.04	3.57	0.12	3.76	0.05	ns	ns	ns
LVID; s (mm)	2.15	0.02	2.38	0.08	2.31	0.03	**	*	ns
IVS; d (mm)	0.75	0.01	0.75	0.01	0.74	0.01	ns	ns	ns
IVS; s (mm)	1.13	0.01	1.06	0.02	1.08	0.01	***	**	ns
LVPW; d (mm)	0.75	0.01	0.75	0.01	0.74	0.01	ns	ns	ns
LVPW; s (mm)	1.13	0.01	1.06	0.02	1.07	0.01	****	***	ns

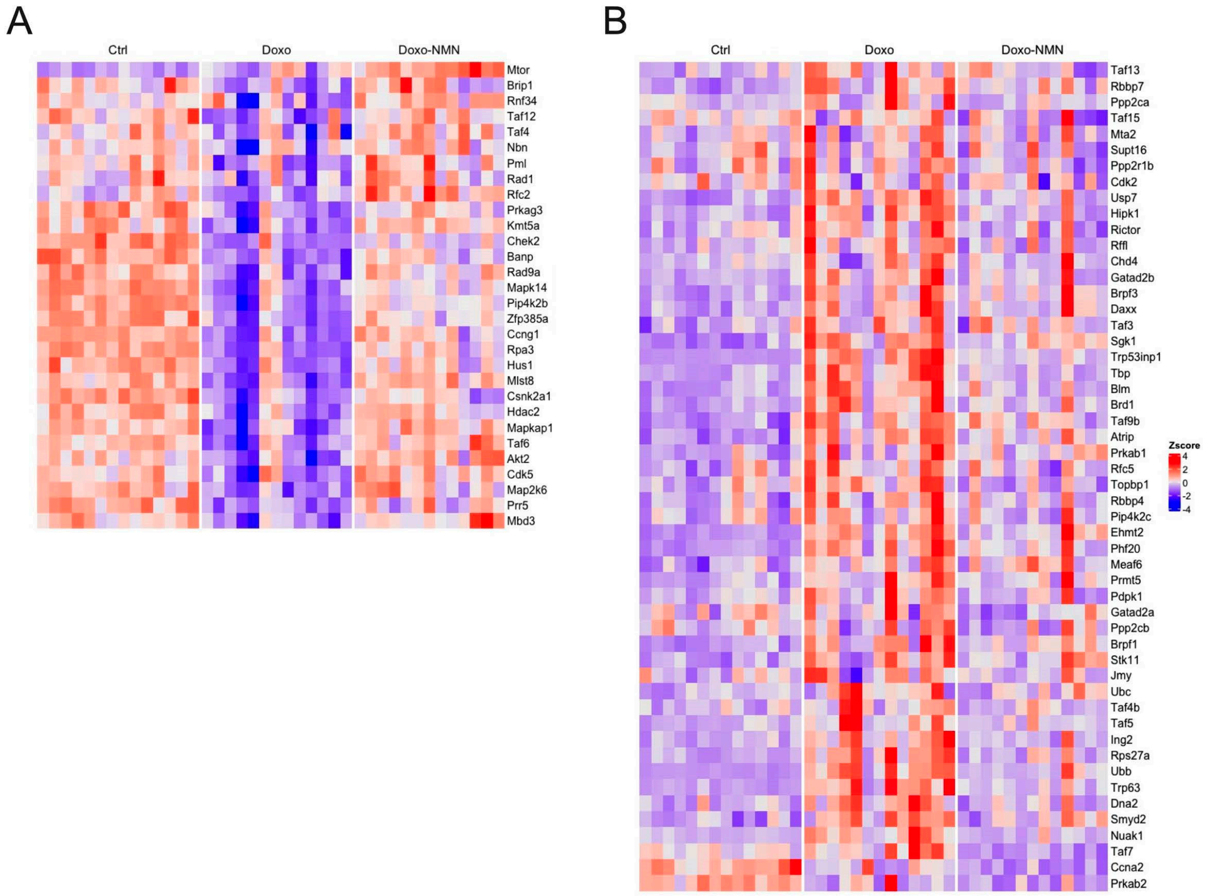

Figure S1: Effect of NMN treatment on regulation of p53 activity in the acute Doxo study. (A-B) Heatmap of genes related to the regulation of p53 activity down-regulated (A) or up-regulated (B) by Doxo vs. Ctrl. Red colors indicate Zscore > 0 (positive regulation) and blue colors indicate Zscore < 0 (negative regulation). Red colors indicate Zscore > 0 (upregulation) and blue colors indicate Zscore < 0 (downregulation).

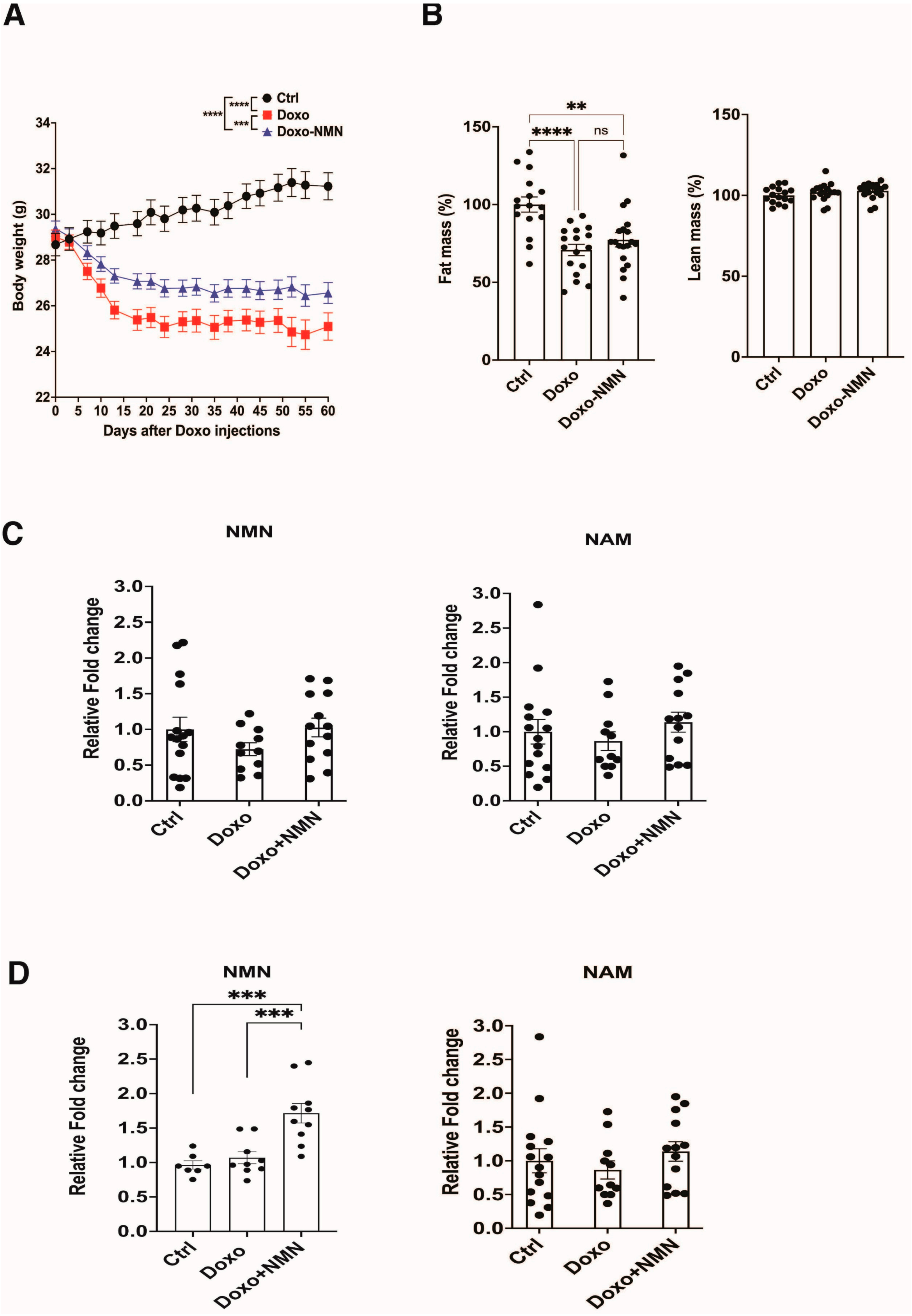

A
B
C
D
Ctrl
Doxo
Doxo-NMN
Body weight (g)
Days after Doxo injections
Fat mass (%)
Lean mass (%)
ns
NMN
NAM
Relative Fold change
Doxo+NMN

Figure S2 The effects of NMN administration on Doxo-induced body weight decrease and NAD-related metabolites in heart and gastrocnemius muscle tissue.

(A) Body weight (grams, g) measurements after the first Doxo injection. One-way Anova followed by Tukey's post-test. ***p < 0.001, ****p < 0.0001.

(B) Body weight composition (percentage, %) at 50 days after the first Doxo injection analyzed by the InAlyzer Dual Energy X-ray Absorption (DEXA) body composition analyzer. Fat mass and lean mass are normalized to the body weight. One-way Anova followed by Tukey's post-test. **p < 0.01, ****p < 0.0001.

(C) Levels of NMN and NAM (relative to Ctrl group) in heart tissue measured by LC-MS/MS.

(D)Levels of NMN and NAM (relative to Ctrl group) in gastrocnemius tissue measured by LC-MS/MS. One-way Anova followed by Tukey's post-test. *p < 0.05, **p < 0.01, ***p < 0.001.

Data are shown as means ± the standard error.
